# SCIB1, a huIgG1 antibody DNA vaccination, combined with PD-1 blockade induced efficient therapy of poorly immunogenic tumors

**DOI:** 10.18632/oncotarget.13070

**Published:** 2016-11-04

**Authors:** Wei Xue, Victoria A. Brentville, Peter Symonds, Katherine W. Cook, Hideo Yagita, Rachael L. Metheringham, Lindy G. Durrant

**Affiliations:** ^1^ Scancell Limited, Academic Department of Clinical Oncology, University of Nottingham, City Hospital, Nottingham, UK; ^2^ Department of Immunology, Juntendo University School of Medicine, Tokyo, Japan; ^3^ Academic Department of Clinical Oncology, University of Nottingham, City Hospital, Nottingham, UK

**Keywords:** Fc targeting, DNA vaccine, PD-1 blockade, melanoma, tumor rejection

## Abstract

**Purpose:**

We have previously shown that supraoptimal signaling of high avidity T cells leads to high expression of PD-1 and inhibition of proliferation. This study was designed to see if this effect could be mitigated by combining a vaccine that stimulates high avidity T cells with PD-1 blockade.

**Experimental Design:**

We investigated the anti-tumor effect of a huIgG1 antibody DNA vaccine (SCIB1) and PD-1 blockade.

**Results:**

Vaccination of HLA-DR4 transgenic mice with SCIB1 induced high frequency and avidity T cell responses that resulted in survival (40%) of mice with established B16F1-DR4 tumors. SCIB1 vaccination was associated with increased infiltration of CD4 and CD8 T cells within the tumor but was also associated with upregulation of PD-L1 within the tumor environment. PD-1 blockade also resulted in increased CD8 T cell infiltration and an anti-tumor response with 50% of mice showing long term survival. In line with our hypothesis that PD-1/PD-L1 signaling results in inhibition of proliferation of high avidity T cells at the tumor site, the combination of PD-1 blockade with vaccination, enhanced the number and proliferation of the CD8 tumor infiltrate. This resulted in a potent anti-tumor response with 80% survival of the mice.

**Conclusions:**

There is a benefit in combining PD-1 blockade with vaccines that induce high avidity T cell responses and in particular with SCIB1.

## INTRODUCTION

Numerous studies have shown that PD-1/PD-L1 pathway is a negative regulator of T cells and that blocking this pathway can lead to improved T cell immunity [1, 2]. Two anti-PD-1 mAbs, Nivolumab and Pembrolizumab have been approved for the treatment of cancer. Nivolumab induces an objective response rate (ORR) of 30-40% in multiple clinical trials in patients with melanoma [3, 4], it extends overall survival in Non-Small-Cell lung cancer [5, 6] and produces an ORR of as high as 87% in Hodgkin's lymphoma [7]. Pembrolizumab has improved efficacy over Ipilimumab [8] and is now approved second line treatment for melanoma. It is also showing encouraging results in NSCLC [9], gastric cancer [10], bladder cancer [11], head and neck cancer [12], Hodgkin's lymphoma [13] and triple negative breast cancer [14]. Although there is evidence that patients whose tumors express PD-L1 have a higher response rate, many PD-L1 negative patients also respond. This discrepancy may be related to “adaptive resistance” which stems from the observation that normal cells and most cell lines do not express PD-L1 unless it is induced by IFNγ. As this cytokine is predominantly secreted by lymphocytes the adaptive theory suggests that tumors are induced to express PD-L1 by tumor infiltrating lymphocytes as a method to limit tissue damage but would also serve to suppress tumor immunity [1]. Thus blockade of the PD-1/PD-L1 interaction will enhance T cell responses at the tumor site thus restricting toxicity. Despite these encouraging responses the majority of patients do not respond to PD-1/PD-L1 blockade. This may be related to the inability of the tumor to stimulate a productive T cell response. Recent studies have suggested that this may be related to the number and/or type of mutations within a tumor which can lead to neo-epitopes [15]. However, non-responders may not have the appropriate mutations and/or fail to stimulate a productive immune response [15, 16]. These patients may benefit from an effective vaccine that stimulates *de novo* high avidity CD4 and CD8 responses prior to checkpoint blockade.

We have previously shown that immunizing with a DNA vaccine incorporating CD8 and CD4 T cell epitopes within an antibody framework (ImmunoBody®) without any additional adjuvants stimulates high frequency, high avidity responses to a wide range of epitopes [17, 18]. The ImmunoBody® acts by direct presentation of the DNA within antigen presenting cells and cross-presentation of secreted protein via the high affinity FcγR1 receptor (CD64). When comparing DNA and protein immunization of ImmunoBody®, the DNA gave higher frequency and avidity responses suggesting direct presentation of the DNA within antigen presenting cells. However, experiments in CD64 knockout mice but not CD32 knockout mice, induced lower frequency and avidity T responses in wild type mice suggesting that cross-presentation of secreted protein via the high affinity FcγR1 receptor (CD64) was important. Although either presentation induces T cell responses, it is only the combination that induces T cells with sufficiently high avidity to kill tumor cells [17, 18]. This was further validated by comparison of the same ImmunoBody® DNA expressing Fab or whole antibody molecules, which showed much weaker responses in the absence of Fc. We have also replaced human IgG1 from the same DNA backbone vector with moIgG2a, both huIgG1 and moIgG2a can stimulate immune responses in mice [17]. SCIB1 is an ImmunoBody® encoding a human IgG1 antibody, with three epitopes from gp100 and one from TRP-2 engineered into its CDR regions. There are two HLA*0201 epitopes, one from TRP-2_180-188_ (SVYDFFVWL) which also is H-2Kb restricted and one from gp100_178-186_ (MLGTHTMEV), and two CD4 epitopes one is HLA-DR4 restricted gp100_44-59_ epitope (WNRQLYPEWTEAQRLD) and the other gp100_174-190_ epitope (TGRAMLGTHTMEVTVYH) restricted by HLA-DR7, HLA-DR53 and HLA-DQ6. We have previously shown that the TRP-2 CD8 epitope breaks tolerance and induces high avidity T cell memory responses to this self-epitope [18]. In this study we show that the gp100DR4 epitope stimulates strong CD4 T cells responses which is consistent with previous publications [17]. A clinical study in stage III/IV melanoma patients with tumor present at study entry showed that SCIB1 could induce T cell responses in 10/11 patients to all 4 encoded epitopes with no associated toxicity. Overall survival was 19 months with patients showing clinical responses including two partial responses and stable disease. Results were even more dramatic in patients with fully resected disease as they all showed a T cell response and are still alive with a current median observation time of 3 years. Two- and three-year recurrence-free survival for stage III resected patients was 89% and 67%, respectively, and for stage IV resected patients it was 71% at both time points [19].

We have shown that potent high avidity T cells induced by SCIB1 express high levels of PD-1 and are susceptible to growth inhibition and apoptosis [20]. If this occurs within the tumor microenvironment due to “adaptive resistance” then a combination of SCIB1 and anti-PD-1 may lead to clinical benefit. We have tested this hypothesis in the poorly immunogenic B16F1-DR4 model using the SCIB1 vaccine. In this study we show that SCIB1 provides efficient tumor therapy in 40% of animals. This can be further enhanced by combination with PD-1 blockade where the therapy promotes proliferation of CD8 T cells within the tumor microenvironment resulting in significantly longer survival in animals than either vaccine or PD-1 blockade alone.

## RESULTS

### Combined SCIB1 and PD-1 blockade induced a strong anti-tumor response

**Figure 1:**
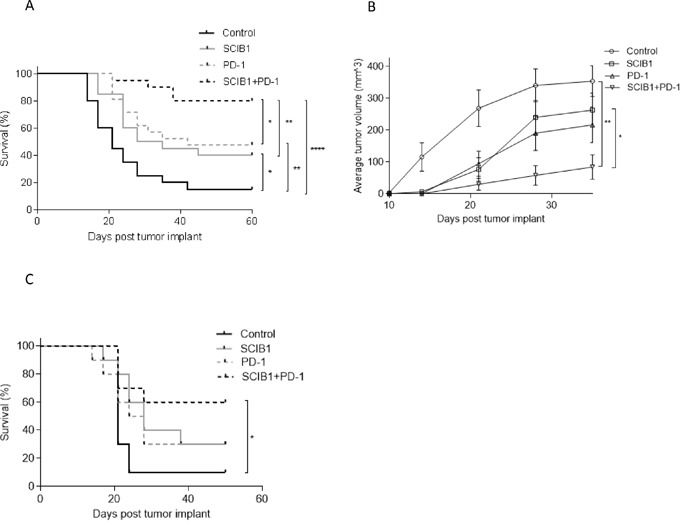
Combination of SCIB1 with PD-1 blockade enhances tumor therapy Mice were challenged with 2.5x10^4^ B16F1-DR4 tumor and immunized with SCIB1, anti-PD-1 antibody or the combination were assessed for **A.** Survival and **B.** Average tumor growth over time. **C.** Survival of mice challenged with 1.5x10^5^ B16F1-DR4 tumors and immunized with SCIB1, anti-PD-1 antibody or the combination. (*, P<0.05; **, P<0.01; ***, P<0.001). Each curve details results from a representative of at least two independent studies with at least 10 mice per group.

We have previously shown that insertion of CD8 epitopes TRP2 (aa180-188) and CD4 epitopes from gp100 (aa44-59 and 174-190) into human IgG1 antibody DNA vaccine (SCIB1) induces high frequency and avidity CD8 and CD4 T cell responses in mice [17] and melanoma patients [19]. In this study, we show that these immune responses result in therapeutic anti-tumor responses against established B16F1-DR4 tumors and in long term survival in 40% of mice (*p*=0.022, Figure 1A). A similar anti-tumor response can be induced by PD-1 blockade which resulted in long term survival in 50% of mice (*p*= 0.0047, Figure 1A). This anti-tumor response could be significantly enhanced by combining PD-1 blockade with SCIB1 showing enhanced survival of 80% compared to control (*p*<0.0001) or when compared to SCIB1 (*p*=0.0066) or PD-1 blockade alone (*p*=0.0234) (Figure 1A). Figure 1B shows growth of tumors was significantly delayed compared to control when mice were treated with SCIB1 (*p*=0.023) or SCIB1 combined with PD-1 blockade (*p*=0.0032). To further investigate the efficacy of combination vaccine, the mice were challenged with a higher dose of B16F1-DR4 tumor cells (1.5x10^5^). Only combination therapy demonstrates a significant survival advantage over control (Control vs SCIB1+PD-1 *p*=0.0126) (Figure 1C).

### SCIB1 induces stronger tumor specific CD8 and CD4 responses than tumor cells whilst anti-PD-1 stimulates a different repertoire

**Figure 2:**
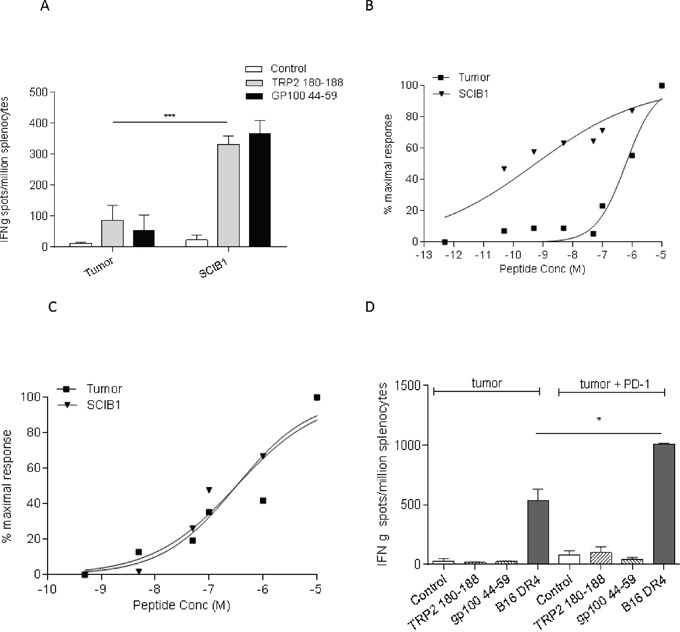
SCIB1 immunization induces better responses compared to tumor **A.** Splenocytes from SCIB1 or tumor immunized mice were analyzed by IFNγ elispot assay for frequency of peptide specific responses. Avidity of responses was measured to increasing TRP2 **B.** or gp100_44-59_ **C.** peptide concentrations. Normalized avidity graphs are shown. **D.** Splenocytes from mice immunized with tumor in the presence or absence of anti-PD-1 Ab were analyzed by IFNγ elispot assay for tumor recognition and frequency of peptide specific responses. Results are representative of at least two independent studies where n≥3. (*, P<0.05; **, P<0.01; ***, P<0.001).

SCIB1 vaccine encodes epitopes from melanoma antigens TRP2 and gp100 which are also present in B16F1-DR4 tumor. The tumor has the potential to stimulate these specific immune responses so analysis was performed to compare the epitope specific immune responses induced by SCIB1 and tumor cells. In HLA-DR4 transgenic mice, SCIB1 induces higher frequency TRP2_180-188_ and gp100_44-59_ epitope specific responses compared to those induced by HLA-DR4 expressing B16 tumor cells (p=0.0002) (Figure 2A). Analysis of the avidity of the responses show that SCIB1 induces higher avidity TRP2 specific responses than those induced by the tumor cells suggesting that tumor provides insufficient stimulation and promotes a weak autoimmune response (Figure 2B). In contrast, both SCIB1 and the tumor induce similar avidity CD4 responses (Figure 2C).

To further understand why PD-1 treatment alone induces anti-tumor responses in our model, we examined its effect on *in vitro* tumor recognition. Interestingly, addition of PD-1 antibody to tumor induced immune responses stimulated better tumor recognition than that seen with immunization with tumor only (Figure 2D). However, the PD-1 blockade did not enhance TRP2 or gp100 specific responses which suggests that PD-1 blockade enhanced responses to tumor epitopes other than TRP2 and gp100.

### Tumor and PD-1 blockade did not influence systemic SCIB1 or tumor induced immune responses

**Figure 3:**
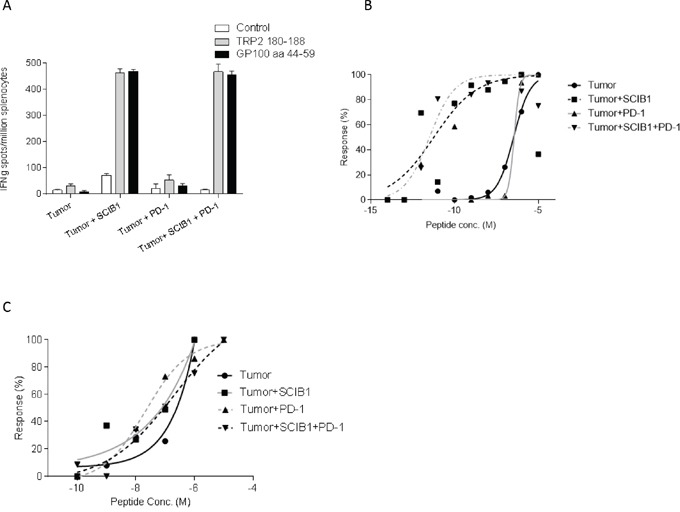
Addition of PD-1 blockade to SCIB1 did not further enhance the immune responses generated in the presence of tumor Mice were implanted with tumor and subsequently vaccinated with SCIB1, PD-1 blockade or combination vaccine. **A.** Splenocytes were assessed by IFNγ elispot assay for frequency of peptide specific responses. Avidity of responses was measured to increasing **B.** TRP2 or **C.** gp100_44-59_ peptide concentrations. Normalized avidity graph is shown. Results are representative of at least two independent studies where n≥3.

Studies examining the adoptive transfer of tumor reactive T cells have demonstrated the induction of exhaustion/tolerance by the tumor. We next sought to determine the effect of tumor on the induction of tumor antigen specific responses in the spleen by SCIB1 and SCIB1 plus PD-1 blockade. Immunization of HLA-DR4 transgenic mice revealed that even in the presence of tumor, SCIB1 still generates high frequency and avidity CD8 and CD4 T cell responses (Figure 3A and 3B). Addition of PD-1 blockade did not alter the frequency or the avidity of the T cell responses induced by vaccination in the presence of tumor suggesting that blockade of PD-1 pathway does not have direct effect on T cell responses generated by SCIB1 (Figure 3A, 3B and 3C). PD-1 blockade also did not significantly enhance the weak CD8 and CD4 responses generated by tumor itself (Figure 3A, 3B and 3C).

### Combined SCIB1 and PD-1 blockade increased CD8 and CD4 T cells within the tumor microenvironment

**Figure 4:**
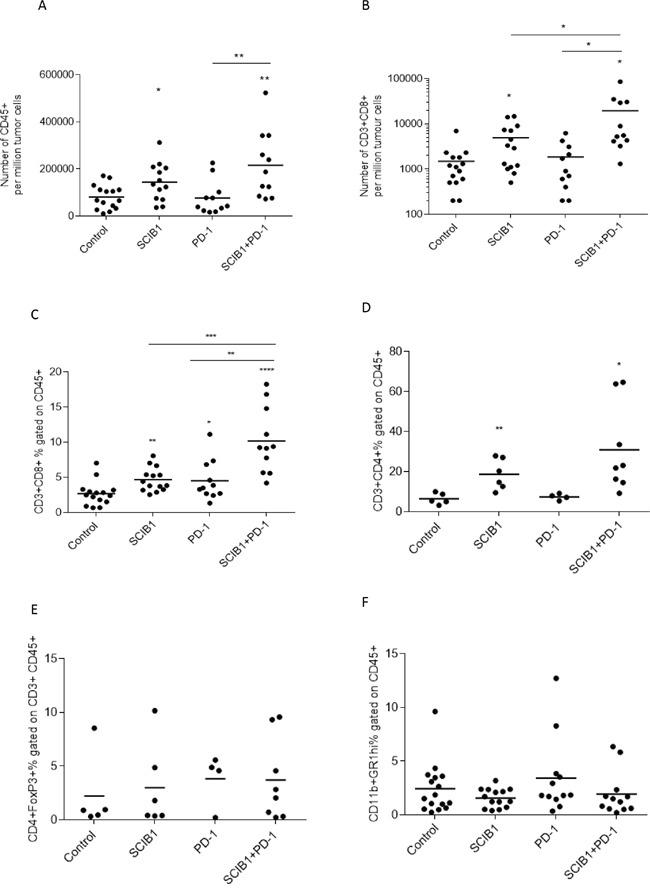
Combination of SCIB1 vaccine with PD-1 blockade increases T cell infiltrate into tumors Tumors from untreated mice or those treated with SCIB1, PD-1 blockade or the combination were assessed for **A.** Number of CD45+ T cell infiltrate per million tumor cells; **B.** Number of CD8+ T cell infiltrate per million tumor cells; **C.** CD3+CD8+ T cell infiltrate as a % of CD45+; **D.** CD3+CD4+ T cell infiltrate as a % of CD45+; **E.** Treg (CD4+FoxP3+) infiltrate as a % of CD45+CD3+; **F.** MDSC (CD11b+GR1^hi^+) infiltrate as a % of CD45+. Analysis of (A) and (B) is gated on the whole tumor cell population. Analysis of (C-F) is gated on CD45+ population while (E) is gated on CD45+CD3+ population (*, P<0.05; **, P<0.01; ***, P<0.001; **** p<0.0001). Results are a combined from at least 2 independent studies in which n≥5.

To further understand the mechanism underlying the strong additive anti-tumor responses, the effects of each treatment on tumor infiltrating lymphocytes was examined. The number of total CD45+ leukocytes was markedly increased following SCIB1 (*p*=0.0192) and SCIB1 plus PD-1 blockade (*p*=0.0024) treatment (Figure 4A). The number of CD3+CD8+ cells was increased 3 fold by SCIB1 vaccination (*p*=0.0157) and was further enhanced 10 fold by the addition of PD-1 blockade (*p*=0.0468) (Figure 4B). When gated on CD45+ population, a similar trend in percentage of CD8 infiltration was seen (Figure 4C). PD-1 blockade alone appeared to have a small effect on the percentage CD8 T cell infiltration (*p*=0.05). The combination vaccine further enhanced CD8 T cells infiltration over PD-1 alone (p=0.0026) or SCIB1 alone (p=0.0004) (Figure 4C). Both SCIB1 alone (*p*=0.0086) and addition of PD-1 blockade to SCIB1 (*p*=0.0321) significantly increased the percentage of CD4 T cells infiltrating the tumor site compared to control (Figure 4D). This suggests that SCIB1 alone and in combination with PD-1 blockade enhances CD8 and CD4 T cell infiltrate into the tumor environment. In contrast, the treatments induced no significant differences in the number of infiltrating Foxp3 expressing regulatory CD4 T cells in the tumors (Figure 4E). Both SCIB1 and SCIB1 plus PD-1 blockade reduced the number of MDSC (CD11b+GR1^hi^+) infiltrating into the tumors but this did not reach significance (Figure 4F).

### PD-1 blockade enhances CD8 T cell proliferation but not T cell migration from the lymph node

**Figure 5:**
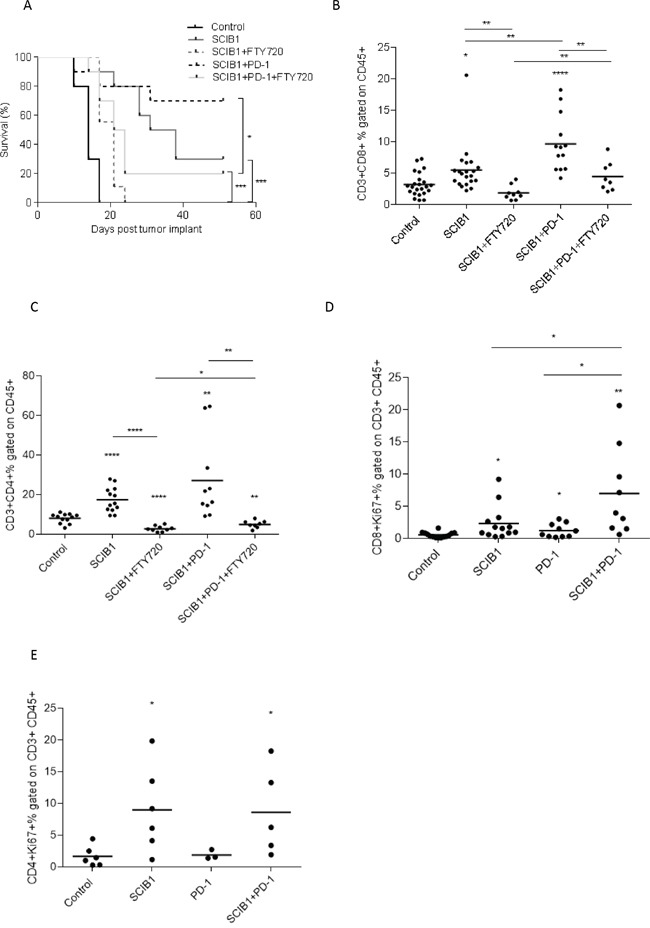
PD-1 blockade enhances CD8 T cell proliferation but not T cell migration from the lymph node Mice were challenged with 1x10^5^ B16F1-DR4 tumor cells and immunized with SCIB1 or combination vaccine in the presence and absence of FTY720 were assessed for **A.** survival, **B.** CD8+ infiltrating T cells, **C.** CD4+ infiltrating T cells. Analysis of (B and C) is gated on CD45+ population. Tumor-infiltrating lymphocytes from mice immunized with SCIB1, PD-1 blockade or combination were assessed for **D.** Ki67 expression on CD8+ T cells and **E.** Ki67 expression on CD4+ T cells. Analysis of (D and E) is gated on CD45+CD3+ population. Results from a representative study with at least 10 mice per group, B-E show combined data from at least 2 independent studies (*, P<0.05; **, P<0.01; ***, P<0.001; ****, p<0.0001).

Since combined therapy showed a strong anti-tumor effect and increased T cell infiltration, we sought to determine if it was a result of further T cell migration from lymph nodes or protection of T cells already at the tumor site. Previous studies showed treatment with FTY720, an immunomodulatory drug, blocks sphingosine 1-phosphate receptors and inhibits T cell migration from lymphoid organs [21, 22]. We therefore utilized it in our anti-tumor model in combination with SCIB1 or SCIB1 plus PD-1 therapy to assess its effect on tumor therapy and T cell infiltration. We showed FTY720 effectively reduced survival of SCIB1 or SCIB1 plus PD-1 treated groups (*P*= 0.001; *P*= 0.0387 respectively; Figure 5A). Analysis of T cell infiltration showed that FTY720 completely blocked the increase in CD8 and CD4 T cell infiltration at tumor site seen with SCIB1 vaccination (Figure 5B and 5C). FTY720 treatment also showed a reduction in T cell infiltrate into tumors in SCIB1 plus PD-1 treated mice. Interestingly, there is a significant difference between FTY720 treated SCIB1 and FTY720 treated SCIB1 plus PD-1 for both CD8 and CD4 infiltration (P=0.0096; P=0.0154) (Figure 5B and 5C). Thus it suggests that although FTY720 could significantly reduce T cell migration into the tumor site induced by SCIB1, PD-1 blockade could still protect the remaining T cells within the tumor resulting in enhanced survival.

T cell proliferation was determined by analyzing Ki67 expression on tumor infiltrating CD8 and CD4 T cells (Figure 5D and 5E). SCIB1 alone (*p*=0.0188) and PD-1 blockade alone (*p*=0.0441) showed significant increase in the number of CD8 T cells expressing Ki67 compared to control (Figure 5D). Addition of PD-1 blockade to SCIB1 resulted in a further increase in the number of CD8 T cells expressing Ki67 compared to SCIB1 alone (*p*=0.0373) or PD-1 blockade alone (*p*=0.0176). There is a small increase in Ki67 expression on CD4 T cells from SCIB1 alone and combination groups (Figure 5E).

### Adaptive resistance

**Figure 6:**
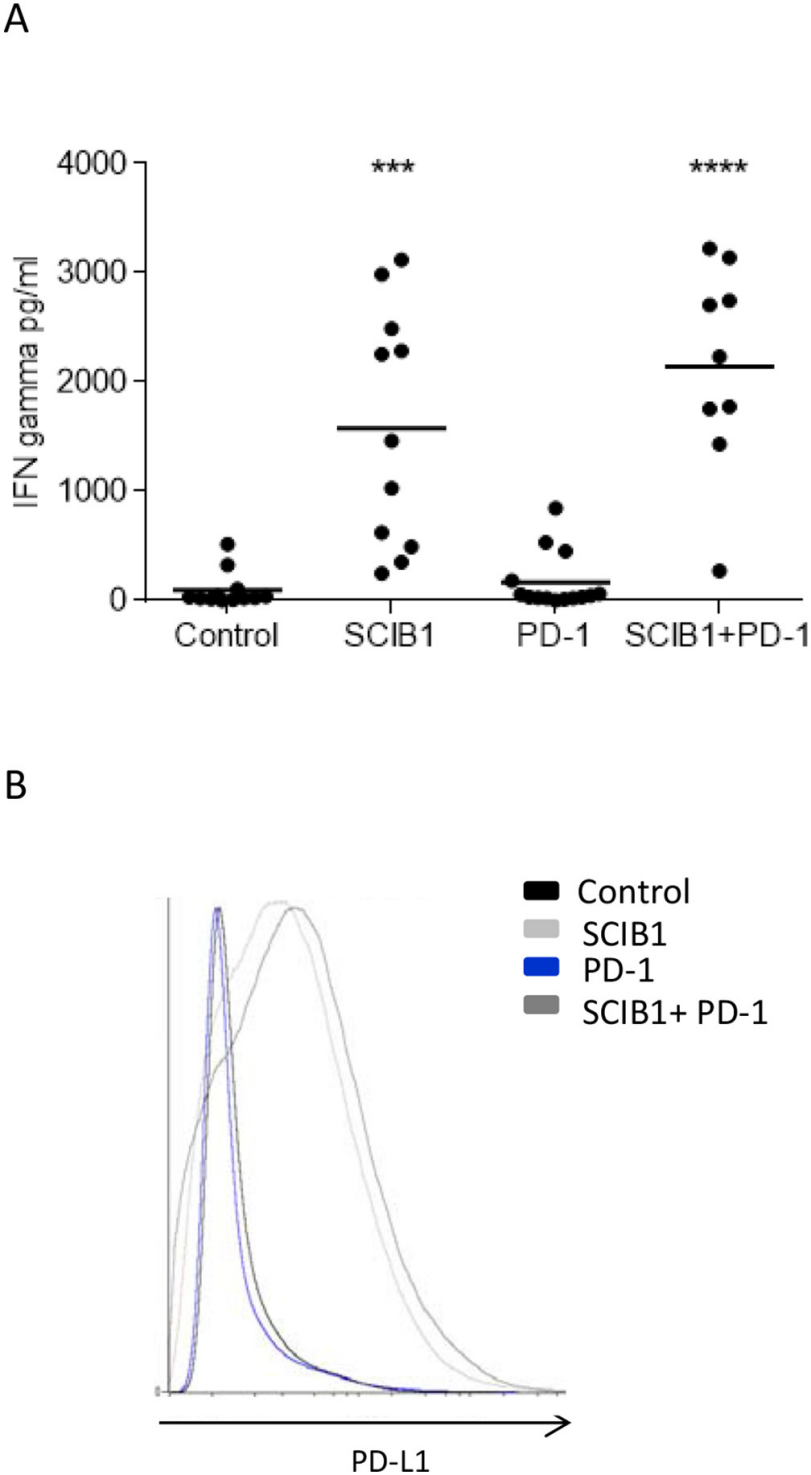
Analysis of TIL function and PD-L1 expression on tumor in mice immunized with single or combination vaccines **A.** TILs from mice immunized with SCIB1, PD-1 blockade or combination were assessed for IFNγ release in response to anti-CD3 stimulation measured by ELISA. Results are combined data from at least two independent studies where n≥5 (***, P<0.001; ****, p<0.0001). **B.** PD-L1 expression within tumor environment where analysis is gated on whole tumor sample and shows representative data n≥3.

PD-L1 is induced on tumor cells by tumor infiltrating lymphocytes releasing IFNγ as a method to limit tissue damage but which also restricts tumor immunotherapy. To determine if the infiltrating lymphocytes were secreting IFNγ, they were isolated and stimulated overnight with anti-CD3 antibody. As demonstrated in Figure 6A, CD3 stimulation results in the production of IFNγ in both SCIB1 alone and combination vaccine groups which are significantly higher than control (*p*=0.0001, *p*<0.0001, respectively) and anti-PD-1 alone (*p*<0.0001; *p*<0.0001, respectively) treated mice. Of particular interest was the low levels of IFNγ produced in the anti-PD-1 alone treated mice, even though they showed a good anti-tumor response. As functional TILs secreting IFNγ could potentially upregulate PDL-1 expression within the tumor microenvironment, levels of PD-L1 on tumors were assessed. In agreement with the TIL results, SCIB1 alone and the combination vaccine upregulated PD-L1 expression whereas control and PD-1 blockade alone did not (Figure 6B).

## DISCUSSION

In this study we demonstrate the efficient induction of high frequency and avidity melanoma antigen specific CD8 and CD4 responses from SCIB1 DNA vaccine. These responses provide tumor therapy in the aggressive murine B16 melanoma model. Thus, suggesting that the high avidity T cell responses, generated by SCIB1 vaccination, where more effective in tumor therapy than the low frequency and avidity T cell responses induced by the tumor itself. SCIB1 therapy was associated with increased infiltration of CD8 T cells within the tumor environment. It is known that high avidity T cells infiltrate more efficiently into tumors and produce cytokines in response to tumor derived antigen [23]. However, the tumor environment can promote dysfunction of high avidity T cells with reduced IFNγ production and expression of CD107a [23, 24]. In this study we have shown that although T cell infiltration resulting from SCIB1 DNA vaccination demonstrates proliferation and cytokine release it was also associated with increased levels of PD-L1 expression in the tumor microenvironment. Several published studies also indicate that the PD-L1 level within the tumor environment can be elevated by infiltrating activated T cells secreting IFNγ [25, 26]. The expansion and function of infiltrating high avidity T cells induced by vaccination could be compromised within the tumor by upregulation of PD-1 and engagement with PD-L1 in the melanoma tumor microenvironment [26–28]. Upregulation of PD-L1 provides another obstacle in the path of vaccine induced anti-tumor T cells. Therefore, the combination of vaccines with blockade of PD-1/PD-L1 signaling may further benefit melanoma patients, especially for late stage disease.

PD-1 blockade as a monotherapy also showed a significant anti-tumor response and an increased infiltration of CD8 but not CD4 T cells. This was likely due to stimulation of a different repertoire of T cells to the SCIB1 vaccine as PD-1 blockade did not induce T cell responses to the TRP2 or gp100 epitopes but did enhance responses to the B16 tumor. These responses may be low avidity as they failed to secrete IFN-γ or upregulate PD-L1 expression on the tumor cells. Studies with adoptive transfer of low avidity T cells failed to control tumor and only when combined with PD-1 blockade resulted in tumor regression [23]. In line with these studies most vaccines that show synergy with PD-1 blockade have not shown regression of established tumors when used alone [29–33] and induce low avidity T cells [30]. Their results demonstrate that it might be possible to rescue a poor vaccine and/or a non-responsive anti-PD-1 tumor by using a combination but then the survival is still very low. In contrast, our vaccine alone gives good survival and anti-PD-1 alone shows therapeutic efficacy in 50% of the animals (similar to results in melanoma patients) but the combination gives an overall survival of 80%. We show additive rather than synergistic responses in the anti-tumor responses as it would take very large numbers of animals to show synergy. We do show synergy with T cell infiltration.

We have demonstrated that the combination of vaccination to induce high avidity tumor specific T cell responses and PD-1 blockade synergises to provide tumor therapy and 85% survival in the aggressive B16 melanoma model. Our previous studies have shown that high avidity CTLs fail to proliferate and express high levels of PD-1 after supraoptimal TCR stimulation in the absence of inflammatory signals [20]. In this study we show that the combination of SCIB1 vaccination to induce high avidity T cells and PD-1 blockade results in a 10 fold increase in infiltration of CD8 T cells due to enhanced proliferation at the tumor site. Data from several studies in different types of cancer have demonstrated that marked infiltration of tumors by immune cells is an independent indicator of good prognosis [34–36]. Anti-PD-1 therapy is known for its localized effect. Analysis of immune responses during PD-1 therapy in clinical trials indicates that the enhancement of T cell function occurs at the tumor site [37]. Our data also suggests that addition of PD-1 blockade to the vaccine is more likely to recover both the vaccine induced and pre-existing responses at tumor site.

In this study we show that SCIB1 and PD-1 blockade are additive resulting in long term survival in 80% of animals. This additive effect of ImmunoBody vaccine and anti-PD-1 antibody is not restricted to these epitopes or DR4 transgenic mice as we have shown similar results with an ImmunoBody targeting NY-ESO-1 in HLA-A2 transgenic mice [38]. We have shown clinical responses in patients receiving SCIB1 treatment alone [19]. Equally melanoma patients receiving anti-PD-1/PD-L1 therapy show response rates of 20-30%. This study would suggest that the combination of SCIB1 and anti-PD-1 should improve this response rate. A new clinical study is being planned. In particular we show that SCIB1 induced IFNγ responses upregulated PD-L1 within the tumor thereby reducing the effectiveness of the vaccine. Studies in lung cancer patients have suggested that anti-PD-1 therapy is more effective in patients whose tumors express high levels of PD-L1 [9]. Thus any vaccine that induces high avidity T cell responses that secrete IFNy within the tumor environment may be expected to show similar additive effects in combination with anti-PD-1 therapy.

## MATERIALS AND METHODS

### Cell lines and media

B16F1 line was obtained from the ATCC and maintained in RPMI with 10% FBS. B16F1-DR4 line was obtained as previously described [39]. Media used for splenocyte culture was RPMI-1640 with 10% fetal bovine serum (FBS), 2mM glutamine, 20mM HEPES buffer, 100 units/ml penicillin, 100μg/ml streptomycin (complete media) and 10^−5^ M 2-mercaptoethanol.

### Reagents

RPMI-1640, FBS, phosphate buffered saline (PBS), penicillin-streptomycin, HEPES, glutamine, 2-mercaptoethanol, collagenase, DNAase and hygromycin B were obtained from Sigma (Poole, UK). Fluorochrome conjugated antibodies targeting CD4 (APC eFluor 780, clone RM4-5), CD3 (PE or PE Cy7, clone 145-2C11), Foxp3 (eFluor 450, clone FJK-16s), Ki67 (FITC, clone SolA15), CD45 (FITC or eFluor 450, clone 30-F11), PD-L1 (PE, clone MIH5), CD3 Ab (purified, clone 17A2), CD11b (APC-eFluor 780, clone M1/70), GR1 (FITC, clone RB6-8C5) were obtained from eBiosciences (San Diego, USA). PE-Alexa-647 labeled antibody targeting CD8 (clone KT15) was obtained from AbD Serotec (Oxford, UK). Anti-PD-1 Ab (clone RMP1-14) was obtained from BioXcell (West Lebanon, USA). Synthetic peptides TRP2_180-188_ (SYVDFFVWL) and gp100_44-59_ (WNRQLYPEWTEAQRLD) were obtained at > 90% purity from Genscript (Piscataway, USA).

### Plasmids

SCIB1 is a plasmid encoding a human IgG1 antibody molecule into which engineered epitopes from overexpressed melanoma antigens have been inserted. Two HLA*0201 epitopes one from TRP-2_180-188_ (SVYDFFVWL) which also is H-2Kb restricted (SVYDFFVW) and one from gp100_178-186_ (MLGTHTMEV) were inserted alongside a HLA-DR4 restricted gp100_44-59_ epitope (WNRQLYPEWTEAQRLD) and a gp100_174-190_ epitope restricted by HLA-DR7, HLA-DR53 and HLA-DQ6 (TGRAMLGTHTMEVTVYH) into the complimentary determining regions of a human IgG1 expressing plasmid as described previously [17].

### Immunization protocol

HLA-DR4 mice (Taconic, USA) were used, aged between 8 and 12 weeks, and cared for by the staff at Nottingham Trent University. All work was carried out under a Home Office approved project license. DNA (1μg/mouse) was coated onto 1.0μm gold particles (BioRad, Hemel Hempstead, UK) using the manufacturer's instructions and administered intradermally by genegun (BioRad, USA). Mice were immunized at day 0, 7 and 14 (unless stated otherwise) and spleens were removed for analysis at day 20.

For tumor challenge experiments mice were challenged with 2.5x10^4^ or 1.5x10^5^ B16F1-DR4 cells subcutaneously on the right flank on day 0 and then were immunized with SCIB1 bullets via genegun i.d. on days 3, 7 and 10. Anti-PD-1 antibody (250μg/dose) was administered i.p. in saline at days 3 and 10. Tumor growth was monitored at 3-4 days intervals and mice were humanely euthanized once tumor reached ≥10 mm in diameter. 200μg/mouse fingolimod (FTY720, Enzo, Exeter, UK) were administrated via intraperitoneal (i.p.) injection 4 hours prior to immunization on days 3, 7, 10 and 14.

### Elispot assay

Elispot assays were performed using murine IFNγ capture and detection reagents according to the manufacturer's instructions (Mabtech, Sweden). In brief, anti- IFNγ antibody was coated onto wells of 96-well Immobilin-P plate. Synthetic peptides (at a variety of concentrations) and 5x10^5^ per well splenocytes were added to the wells of the plate in triplicate. Plates were incubated for 40hrs at 37°C. After incubation, captured IFNγ was detected by biotinylated anti- IFNγ antibody and developed with a strepatavidin alkaline phosphatase and chromogenic substrate. Functional avidity was calculated as the concentration mediating 50% maximal effector function using a graph of effector function versus peptide concentration.

### Tumor infiltrate analysis

Tumors were harvested, mechanically disaggregated and digested with 1mg/ml collagenase and 100 μg/ml DNase and incubated for 30 minutes (Sigma, USA). Cells were stained with antibodies targeting cell surface and intracellular antigens.

For functional infiltrate analysis tumors were mechanically disaggregated and passed through a 0.4μM cell strainer (Falcon, UK). Remaining cells were counted and plated into 96 well plates previously coated with anti-CD3 antibody (eBioscience, USA). Plates were incubated for 20hrs and supernatant assessed for IFNγ release by ELISA using kits from Mabtech according to manufacturer's instructions.

### Statistics

Comparative analysis of the Elispot, flow cytometry and tumor volumes data was performed by applying the student's t-test with values of p calculated accordingly. Comparison of avidity curves was performed by applying two-way ANOVA and survival analysis by applying the Log-rank test using Graphpad Prism software.
